# Recurrent Clostridioides difficile Infection in a Patient With Chronic Colitis: A Successful Response to Fecal Microbiota Transplantation

**DOI:** 10.7759/cureus.88285

**Published:** 2025-07-19

**Authors:** Sally Hamad, Beebee Mubarak Jan, Abdulrahman Al-Mohammed

**Affiliations:** 1 General Medicine, Peterborough City Hospital, Peterborough, GBR; 2 General Surgery, King's College Hospital, London, GBR; 3 General Medicine, Novosibirsk State University, Novosibirsk, RUS

**Keywords:** fecal microbiota transplantation (fmt), gastrointestinal pathology, multiple sclerosis, recurrent clostridioides difficile infection, sarcoidosis

## Abstract

Recurrent *Clostridioides difficile* infection (rCDI) remains a significant treatment challenge, particularly in patients with underlying gastrointestinal conditions. We present the case of a 72-year-old woman with multiple sclerosis and sarcoidosis, who experienced four separate episodes of rCDI despite treatment with vancomycin, fidaxomicin, and intravenous metronidazole. Colonoscopy revealed patchy inflammation with aphthous ulcerations, and histology confirmed chronic colitis without dysplasia or cytomegalovirus infection. Following a structured vancomycin taper, the patient underwent fecal microbiota transplantation (FMT), with complete resolution of symptoms. This case supports the early use of FMT in rCDI and highlights the need for individualized treatment strategies in patients with co-existing colonic inflammation.

## Introduction

*Clostridioides difficile* infection (CDI) is a significant cause of antibiotic-associated diarrhea and colitis, especially in older adults and hospitalized patients. The pathogenesis involves antibiotic-induced disruption of the gut microbiota, allowing overgrowth of toxigenic *C. difficile* strains. While first-line treatments such as vancomycin and fidaxomicin are often effective, approximately 20%-30% of patients experience recurrent infection after initial resolution [1]. The risk of further recurrence increases with each subsequent episode, leading to cumulative morbidity, prolonged hospitalization, and reduced quality of life [2].

Management of recurrent CDI (rCDI) is particularly challenging in patients with concurrent gastrointestinal disorders such as inflammatory bowel disease (IBD) or chronic colitis. In such cases, it may be difficult to distinguish between infectious and inflammatory etiologies of diarrhea. Furthermore, the repeated use of antibiotics can worsen gut dysbiosis, potentially exacerbating both infection and inflammation [3].

Fecal microbiota transplantation (FMT) has emerged as a highly effective treatment for rCDI, especially in patients who have failed multiple courses of antibiotics. The goal of FMT is to restore microbial diversity and re-establish colonization resistance against *C. difficile*. Clinical trials and meta-analyses have reported success rates exceeding 85% in preventing further recurrence [4,5]. FMT is now recommended by several international guidelines for patients with multiple CDI relapses [1].

In this report, we present the case of an elderly patient with rCDI and co-existing chronic colitis who failed standard antibiotic therapies but responded successfully to FMT. The case highlights the diagnostic complexity of rCDI in patients with underlying bowel pathology and underscores the importance of early multidisciplinary intervention and timely escalation to microbiota-based therapy.

## Case presentation

A 72-year-old woman with a past medical history of multiple sclerosis and sarcoidosis was admitted in December 2024 with 8 to 10 episodes of watery diarrhea per day, associated with mild lower abdominal discomfort and no overt rectal bleeding or mucus. She remained apyrexial and hemodynamically stable, with no signs of peritonism. A stool sample tested positive for *C. difficile* toxin, and she was treated with a 10-day course of oral vancomycin. Her symptoms initially improved, but she re-presented in January 2025 with recurrent symptoms. A second stool sample again tested positive for *C. difficile* toxin. She was switched to fidaxomicin and discharged with clinical improvement.

In February 2025, she experienced a third episode of diarrhea. She had not received antibiotics in the interim. She was retreated with fidaxomicin, but her symptoms persisted intermittently. A fourth recurrence occurred in April 2025, for which she was admitted and treated with oral vancomycin and intravenous metronidazole. Despite multiple antibiotic regimens, her diarrhea continued with limited intervals of improvement between episodes.

**Figure 1:**
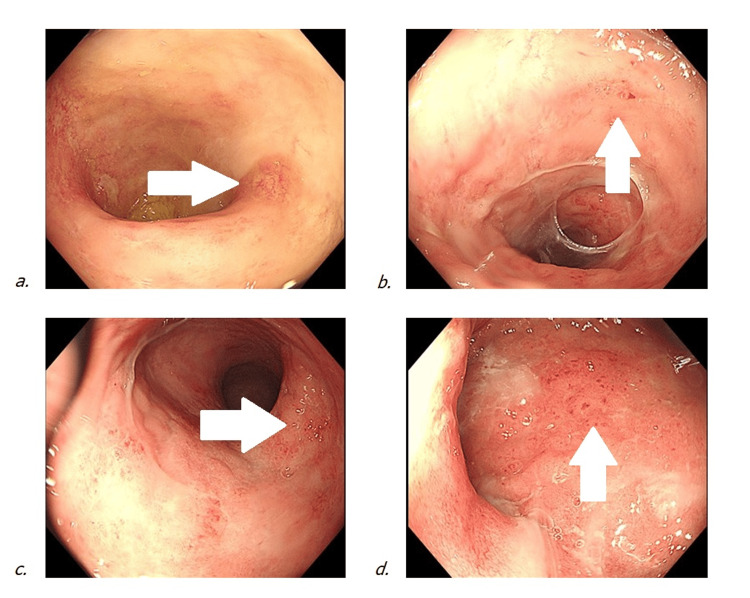
Endoscopic Images From March 2025 Showing Moderate Active Colitis (a) Ulcerated and inflamed mucosa at the splenic flexure with loss of vascular pattern and mucosal friability (white arrow). (b) Distal descending colon showing mucosal erythema and early aphthous ulceration (white arrow). (c) Proximal sigmoid colon with patchy erythema and granularity, consistent with moderate colitis (white arrow). (d) Rectum demonstrating diffuse mucosal inflammation and scattered ulcerations (white arrow).

A flexible sigmoidoscopy performed in March 2025 showed moderate active inflammation extending up to the splenic flexure (Figure 1a), with mucosal ulceration throughout the rectum (Figure 1d), sigmoid colon (Figure 1c), and descending colon (Figure 1b). Multiple biopsies were taken. Histological analysis demonstrated distorted crypt architecture, patchy chronic inflammation, and prominent eosinophils and plasma cells in the lamina propria. There was no evidence of crypt abscesses, granulomas, dysplasia, or cytomegalovirus (CMV) infection. These findings were consistent with chronic colitis.

**Figure 2:**
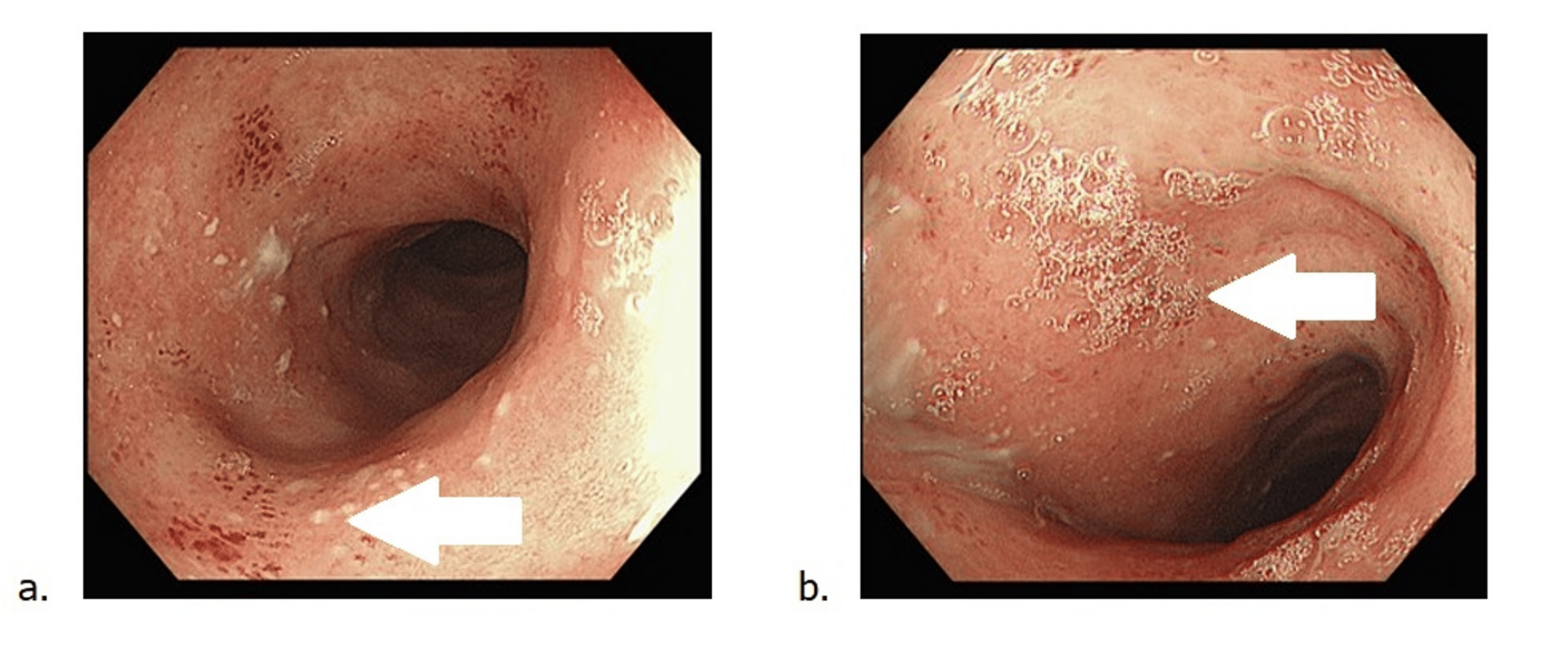
Endoscopic Images From May 2025 Demonstrating Improving Colitis With Patchy Inflammation (a) Rectum showing improved mucosa with scattered aphthous ulcerations and patchy loss of vascular pattern (white arrow). (b) Sigmoid colon with mild residual erythema and surface granularity, suggestive of healing colitis (white arrow).

A repeat sigmoidoscopy in May 2025 revealed patchy loss of vascular pattern, scattered aphthous ulcerations, and intervening areas of normal mucosa (Figure 2), suggestive of healing disease. Biopsies again confirmed chronic colitis without dysplasia or CMV.

**Figure 3:**
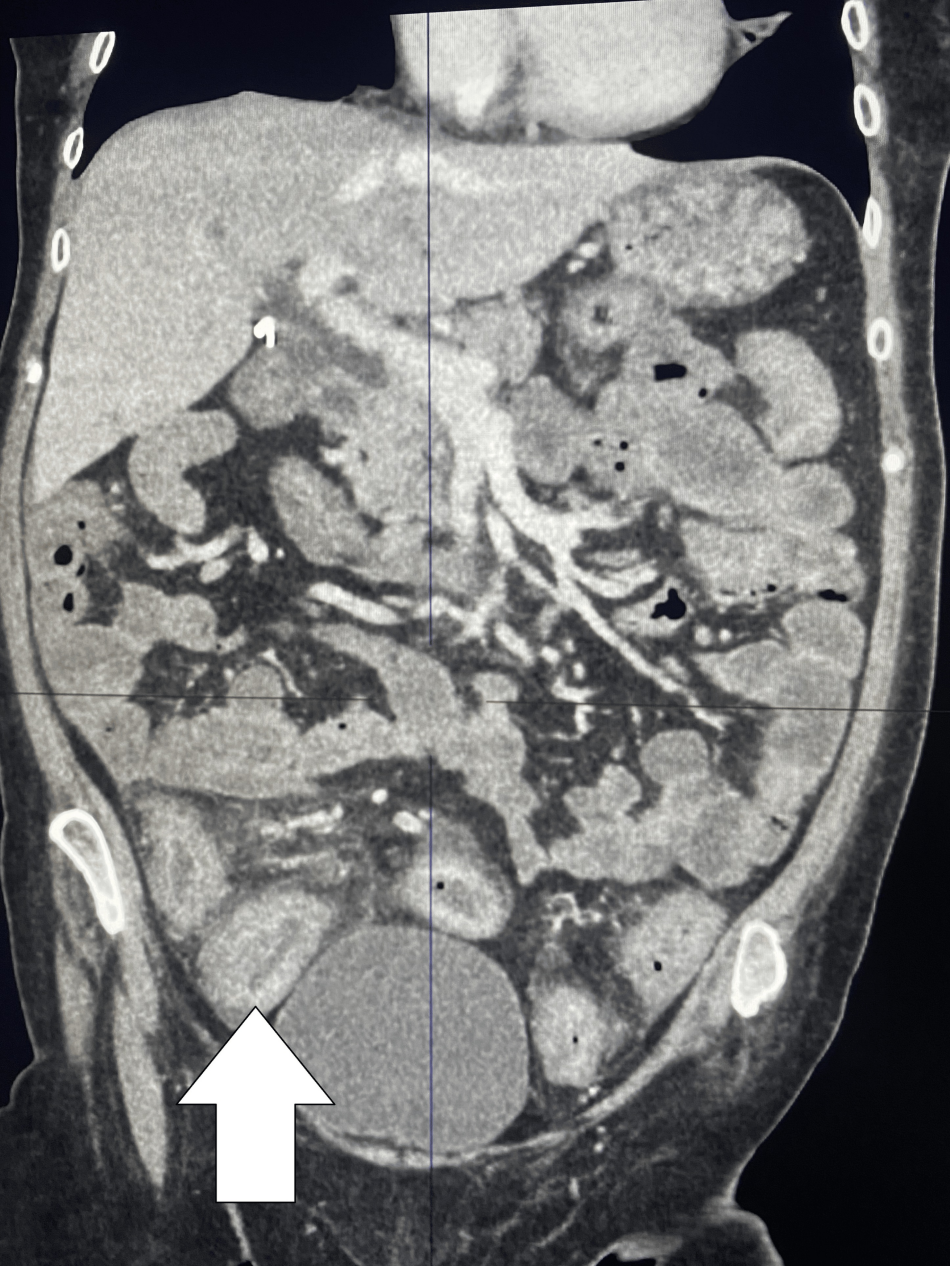
CT Abdomen and Pelvis From March 2025 Showing Active Colitis Contrast-enhanced coronal CT image demonstrating diffuse colonic wall thickening and edema. The white arrow highlights a segment of notably thickened bowel.

A contrast-enhanced CT of the abdomen and pelvis was performed in March 2025, due to a CRP exceeding 300 mg/L and clinical concern for toxic colitis. The scan showed diffuse colonic wall thickening and edema, with no signs of perforation, ischemia, or fluid collections. There was also mesenteric edema and small-volume free fluid in the paracolic gutters (Figure 3). These findings were consistent with ongoing colitis without radiological complications.

In consultation with the infectious diseases and gastroenterology teams, a decision was made to proceed with FMT. The primary goal was to eradicate *C. difficile* spores, restore microbiome diversity, and prevent further recurrences after multiple failed antibiotic treatments. The patient completed a six-week vancomycin tapering regimen before the procedure. FMT was administered via nasogastric tube on June 5, 2025, using material processed according to the Birmingham protocol. The patient was kept nil by mouth for six hours before the procedure and received domperidone as a prokinetic agent prior to FMT.

The procedure was well tolerated, with no vomiting, abdominal pain, fever, or hemodynamic instability observed during or in the hours following nasogastric FMT administration. Within days, the patient reported resolution of diarrhea and return to normal bowel habits. No adverse effects were noted. She remained clinically well and was discharged home with outpatient follow-up. At the one-month review, the patient reported complete resolution of diarrheal symptoms. Stool toxin assays remained negative, and no further admissions were required. No recurrence of CDI was observed following FMT.

## Discussion

rCDI presents a growing clinical burden, particularly in elderly or immunocompromised patients. In this case, the patient experienced four documented episodes over five months, each confirmed by positive toxin testing and consistent clinical features. Despite appropriate courses of vancomycin and fidaxomicin, she continued to relapse, reflecting a treatment-refractory course.

Diagnosis was further complicated by underlying colonic inflammation. Endoscopic and histological findings supported chronic colitis, though they were not typical for ulcerative colitis or Crohn’s disease. The presence of patchy mucosal ulceration and persistent crypt architectural distortion without active cryptitis or granulomas pointed toward post-infectious or inflammatory changes. CMV colitis was ruled out through immunohistochemical staining, and radiologic imaging corroborated the presence of diffuse colitis, but no complications such as perforation or toxic megacolon were identified.

Multiple studies have demonstrated the efficacy of FMT in patients with rCDI, particularly after two or more failed antibiotic courses [2]. FMT works by restoring microbial diversity and displacing toxigenic strains of *C. difficile*. Its efficacy has been reported to exceed 85% in appropriately selected patients [1,2]. In this case, a structured vancomycin taper followed by nasogastric FMT led to full resolution of symptoms, and no further recurrence, consistent with outcomes reported in the literature [3].

Clinical guidelines from the Infectious Diseases Society of America (IDSA) and the European Society of Clinical Microbiology and Infectious Diseases (ESCMID) now recommend FMT as a therapeutic option in patients with multiple recurrences of CDI who have failed standard therapy [4,5]. This case reinforces the need to escalate treatment early in patients at risk of ongoing recurrence and demonstrates the value of combining endoscopic, histologic, and radiologic evaluation in guiding clinical decision-making.

## Conclusions

This case highlights the clinical complexity of managing rCDI in the context of chronic colonic inflammation. The patient presented with four documented relapses over a four-month period, despite receiving multiple antibiotic regimens, including oral vancomycin, fidaxomicin, and intravenous metronidazole. Imaging revealed diffuse colonic wall thickening and edema, while endoscopy showed patchy mucosal changes, and histopathology ruled out IBD or CMV colitis. In consultation with the infectious diseases and gastroenterology teams, FMT was performed via nasogastric tube following tapering vancomycin. The procedure was well tolerated, and no adverse effects were observed. The patient remained symptom-free during outpatient follow-up, with no further recurrence of CDI. In patients who fail multiple antibiotic regimens, FMT should be considered early. Careful assessment through endoscopy, imaging, and histopathology is essential to exclude other pathologies and support appropriate use of FMT. This case adds to the growing body of evidence supporting FMT as a safe, effective, and durable treatment strategy for recurrent CDI.
